# Cost-Effectiveness of Nivolumab Plus Ipilimumab as First-Line Therapy in Advanced Non–small-cell Lung Cancer

**DOI:** 10.3389/fphar.2021.573852

**Published:** 2021-07-05

**Authors:** Xuezhi Hao, Aizong Shen, Bin Wu

**Affiliations:** ^1^ Department of Medical Oncology, National Cancer Center/National Clinical Research Center for Cancer/Cancer Hospital, Chinese Academy of Medical Science and Peking Union Medical College, Beijing, China; ^2^ Department of Pharmacy, The First Affiliated Hospital of University of Science and Technology of China, Hefei, China; ^3^ Medical Decision and Economic Group, Department of Pharmacy, Ren Ji Hospital, South Campus, School of Medicine, Shanghai Jiaotong University, Shanghai, China

**Keywords:** ipilimumab, nivolumab, advanced non-small-cell lung cancer, chemotherapy, cost-effectiveness

## Abstract

**Purpose:** The effectiveness of nivolumab plus ipilimumab for advanced non-small cell lung cancer (NSCLC) has been demonstrated. Decisions have to be made about allocating healthcare resources. Economic evidence could support policy decisions to fund expensive interventions. The current analysis evaluated the cost-effectiveness of nivolumab plus ipilimumab in advanced NSCLC harboring no EGFR or ALK mutations. It is set in the context of the US and China, representing developed and resource-constrained settings, respectively.

**Patients and Methods:** A Markov model consisting of three discrete health states was used to assess the cost-effectiveness of nivolumab plus ipilimumab vs. chemotherapy. The key clinical data were derived from the CheckMate-227 trial, and the cost and health preference data were derived from the literature. Costs, quality-adjusted life-years (QALYs), incremental cost-effectiveness ratios (ICERs) and incremental net health benefits (INHBs) were calculated for the two strategies. Subgroup, one-way and probabilistic sensitivity analyses were performed.

**Results:** In the United States, nivolumab plus ipilimumab increased by 1.260 QALYs with an additional cost of $95,617 compared with the features of chemotherapy, which led to an ICER of $75,871 per QALY gained. INHB indicated that nivolumab plus ipilimumab treatment had a 99% probability of being cost-effective at the ICER threshold of $100,000/QALY in all subgroups. The results of sensitivity analyses revealed that the model outcomes were robust. In China, the ICER of nivolumab plus ipilimumab vs. chemotherapy was $59,773/QALY, and the INHB was -1.972 QALY at the threshold of $27,351/QALY.

**Conclusion:** Nivolumab plus ipilimumab treatment is a cost-effective option compared with chemotherapy for patients with advanced NSCLC harboring no EGFR or ALK mutations in the United States. However, nivolumab plus ipilimumab is not a preferred option in China.

## Introduction

The Global Burden of disease Study revealed that lung cancer is one of the leading causes of non-communicable disease worldwide (The Global Burden of Disease Study, 2019). Approximately 85–90% of lung cancers are non-small-cell lung cancer (NSCLC). Platinum-based chemotherapy has been the standard of care for the first-line treatment of metastatic NSCLC without EGFR or ALK mutations (Griesinger et al., 2019). However, the overall survival (OS) and progression-free survival (PFS) of chemotherapy are unsatisfactory with metastatic NSCLC.

Recently, the use of immune checkpoint inhibitors (ICIs) as a treatment for blocking the programmed cell death one ligand 1 (PD-L1) and programmed cell death 1 (PD-1) pathways has become standard as a replacement for chemotherapy (Zhou et al., 2018; Gubens and Davies, 2019; Peters et al., 2019). Nivolumab, a fully human anti-PD-1 antibody, and ipilimumab, a fully human anti-cytotoxic T-lymphocyte antigen 4 (CTLA-4) antibody, are immune checkpoint inhibitors with distinct but complementary mechanisms of action. The phase three CheckMate-227 trial showed that first-line treatment with nivolumab plus ipilimumab prolonged the median overall survival of 3.2 months in comparison with chemotherapy in patients with advanced NSCLC (Hellmann et al., 2019). However, due to the prohibitive cost of implementing nivolumab plus ipilimumab in the first-line setting, the cost-effectiveness of nivolumab plus ipilimumab needs to be evaluated. The present analysis investigated the economic outcomes of implementing nivolumab plus ipilimumab regimens for treating newly diagnosed advanced NSCLC in the first-line setting from the United States third-party payer and Chinese health care perspectives, representing developed and resource-constrained settings, respectively.

## Materials and Methods

### Model Structure

**FIGURE 1:**
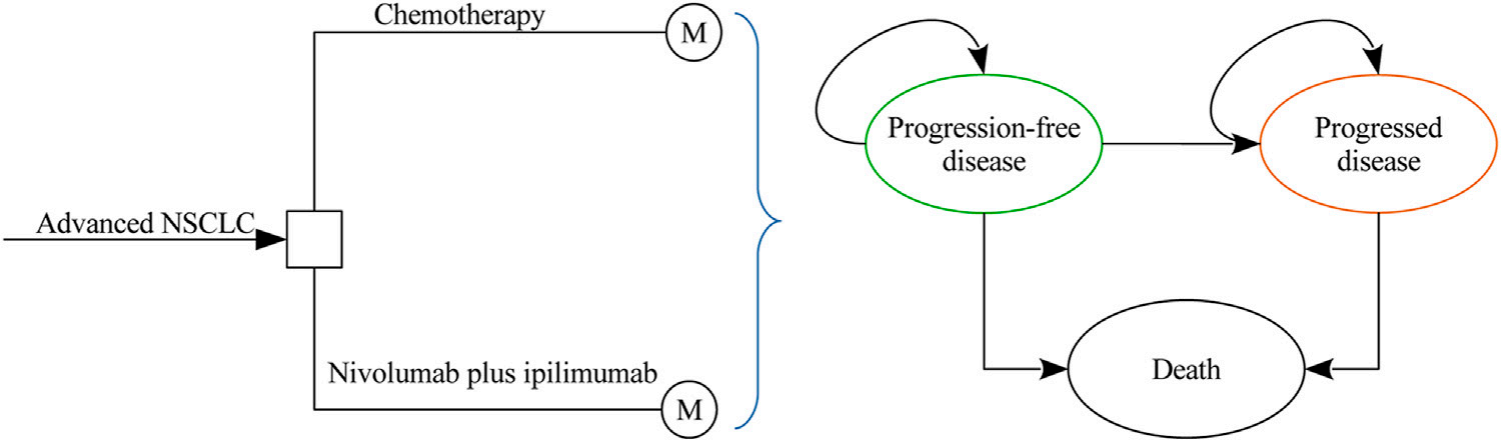
Model structure.

A Markov model was developed to evaluate the costs and health outcomes of treating advanced NSCLC with chemotherapy and nivolumab plus ipilimumab. The model included three discrete health states reflecting different characteristics of the disease: progression-free disease (PFD), progressed disease (PD), and death (Figure 1). The cycle length of the Markov model was one week with a 10 years time horizon, and the initial health state for all of the patients was PFD. The 10 years time horizon was adopted because the long-term survival of patients with advanced NSCLC is still uncertain in current clinical practice. During each one-week cycle, the patients either remained in their assigned health state or progressed to a new health state. The hypothetical patient demographics when entering the model matched those of the patients in the CheckMate-227 trial (Hellmann et al., 2019): previously untreated squamous or nonsquamous stage IV or recurrent NSCLC without EGFR or ALK mutations. Model development and data analysis were performed in the R statistical environment (version 3.4.2; R Development Core Team, Vienna, Austria). The current analysis was carried out from the US third-party payer and Chinese health care perspectives, which means that the two scenarios shared the same clinical and utility inputs except the local cost estimates and life table data.

### Clinical Model Inputs

The PFS and OS data for chemotherapy and nivolumab plus ipilimumab were informed by the results of the CheckMate-227 trial (Hellmann et al., 2019). The virtual patient-level data were reconstructed by using standard statistical analyses described by Guyot et al. (2012). The digitized R package (https://github.com/tpoisot/digitize/) was used to gather the data points from the PFS and OS curves, and these data points were then used to fit the following parametric survival functions for exploring the survival probabilities over the model time horizon: Weibull, log-normal, log-logistic, exponential, Gompertz, Royston/Parmar spline model, mixture and non-mixture cure models. The Akaike information criterion was used to measure the goodness of fit. In the full cohort with unknown PD-L1 status, the Royston/Parmar spline and log-logistic models were found to be the most rational models to extrapolate the long-term PFS and OS of chemotherapy, and the Royston/Parmar spline model and the mixture cure model with a Gompertz distribution were used for nivolumab plus ipilimumab, respectively. The model parameters are shown in Table 1. The constructed patient-level data were generated from Kaplan–Meier curves by using event and censor times, which closely matched the reported Kaplan–Meier curves of the CheckMate-227 trial (Hellmann et al., 2019). The PFS and OS Kaplan–Meier graphs generated by using the constructed data and the predicted curves by adopting the selected parametric survival models are presented in Supplementary Appendix Figures S1, S2. By using the fitted PFS and OS parametric survival models, denoted as P(t) and S(t), the transition probability Prob_*(PFS→PD)*_ and cancer-specific mortality Prob_*(PD→Death)*_ at cycle t were computed as Prob_*(PFS→PD)*_ = (P_[t]_ − P_[t+1]_)/P_(t)_ and Prob_*(PD→Death)*_ = (S_[t]_ − S_[t+1]_)/(S_[t]_ − P_[t]_), respectively. After the cancer progressed, the proportions of patients who received subsequent active therapy were extracted from the CheckMate-227 trial (Hellmann et al., 2019). By considering long-term survival, all-cause mortality estimated from United States Life Tables (2015) was adopted beyond the observation period of the CheckMate-227 trial (Hellmann et al., 2019). The Chinese lifetable was extracted from the Global Health Observatory data (WHO Life tables, 2020).

**TABLE 1:** Model parameters: Baseline values, ranges, and distributions for sensitivity analysis.

Paramters	Expected value	Range	Distribution	References
Clinical inputs				Hellmann et al. (2019)
Survival model of chemotherapy				
Royston/Parmar spline model for PFS	gamma0 = −5.8857; gamma1 = 1.7872; gamma2 = −0.4154; gamma3 = 0.5401 (AIC = 3,793.24)			
Log-normal model for OS	Shape = 1.484; scale = 58.3458 (AIC = 4,976.50)			
Survival model of nivolumab plus ipilimumab				
Royston/Parmar spline model for PFS	gamma0 = −5.8042; gamma1 = 3.4166; gamma2 = 0.2004; gamma3 = −0.1254 (AIC = 4,033.88)			
Mixture cure model with gompertz distribution for OS	Theta = 0.3156; shape = 0.005; rate = 0.0148 (AIC = 4,375.30)			
Proportion of receiving subsequent treatment				
Chemotherapy	0.56	0.422–0.704	Beta: α= 7, β= 5.4	
Nivolumab plus ipilimumab	0.43	0.321–0.535	Beta: α= 9.2, β= 12.2	
Probability of AEs				
Grade ≥3 AEs in chemotherapy	0.36	0.27–0.45	Beta: α= 10.2, β= 18.2	
Grade ≥3 AEs in nivolumab plus ipilimumab	0.33	0.246–0.41	Beta: α= 10.8, β= 22	
Utility inputs (time to death in days)				(Insinga et al., 2018; Insinga et al., 2019)
≥360	0.84	0.823–0.861	Beta: α= 1,192, β= 223.7	
[180, 360)	0.81	0.795–0.833	Beta: α= 1,311.5, β= 299.7	
[30,180)	0.74	0.717–0.756	Beta: α= 1,443.2, β= 515	
<30	0.57	0.481–0.655	Beta: α= 70.7, β= 53.8	
Cost inputs in the United States				
Ipilimumab per 50 mg	7,656	3,828–7,656	Fixed	CMS (2019)
Nivolumab per 100 mg	2,781	1,390–2,781	Fixed	CMS (2019)
Platium-doublet chemotherapy per patient/four 3 weeks chemotherapy cycles	24,437	18,328–30,547	Gamma: α= 190,916, λ= 0.128	Yu et al. (2018)
Maintenance chemotherapy with pemetrexed per 3 weeks cycle	5,887	4,415–7,359	Gamma: α= 45,994, λ= 0.128	Yu et al. (2018)
Post-discontinuation treatment in nivolumab plus ipilimumab treatment	13,097	9,823–16,371	Gamma: α= 52,388, λ= 0.25	Insinga et al. (2018); Insinga et al. (2019)
Post-discontinuation treatment in standard chemotherapy treatment	41,161	30,871–51,451	Gamma: α= 164,644, λ= 0.25	Insinga et al. (2018); Insinga et al. (2019)
disease management in PFD state per one-week in 1st year	1,313	984–1,641	Gamma: α= 5,252, λ= 0.25	Insinga et al. (2018); Insinga et al. (2019)
disease management in PFD state per one-week in 2nd year	696	522–870	Gamma: α= 2,784, λ= 0.25	Insinga et al. (2018); Insinga et al. (2019)
disease management in PFD state per one-week in 3rd year	307	230–384	Gamma: α= 1,223, λ= 0.251	Insinga et al. (2018); Insinga et al. (2019)
disease management in PFD state per one-week in 4th to 5th year	255	191–319	Gamma: α= 1,016, λ= 0.251	Insinga et al. (2018); Insinga et al. (2019)
disease management in PFD state per one-week in after 5 years	112	84–140	Gamma: α= 448, λ= 0.25	Insinga et al. (2018); Insinga et al. (2019)
disease management in PD state per one-week in 1st year	1,448	1,086–1811	Gamma: α= 5,792, λ= 0.25	Insinga et al. (2018); Insinga et al., 2019)
disease management in PD state per one-week in 2nd year	1,015	761–1,268	Gamma: α= 4,060, λ= 0.25	Insinga et al. (2018); Insinga et al. (2019)
disease management in PD state per one-week in 3rd year	858	644–1,073	Gamma: α= 3,418, λ= 0.251	Insinga et al. (2018); Insinga et al. (2019)
disease management in PD state per one-week in 4th to 5th year	818	613–1,022	Gamma: α= 3,285, λ= 0.249	Insinga et al. (2018); Insinga et al. (2019)
disease management in PD state per one-week in after 5 years	818	613–1,022	Gamma: α= 3,285, λ= 0.249	Insinga et al. (2018); Insinga et al. (2019)
Managing AE (grade ≥3) per patient related to ICI treatment	1,499	1,124–1874	Gamma: α= 5,996, λ= 0.25	Insinga et al. (2018); Insinga et al. (2019)
Managing AE (grade ≥3) per patient related to chemotherapy	1,259	944–1,574	Gamma: α= 5,036, λ= 0.25	Insinga et al. (2018); Insinga et al. (2019)
Terminal care (last 30 days of life)	15,498	11,624–19,373	Gamma: α= 61,992, λ= 0.25	Insinga et al. (2018); Insinga et al. (2019)

Abbreviations: AE, adverse event; PFS, progression-free survival; PFD, progression-free disease; PD, progressed disease; OS, overall survival

### Cost and Utility Model Inputs

The current analysis was carried out from Unites States third-party payer and Chinese health care perspectives. Therefore, only direct medical costs were considered in this analysis, including the drug costs, laboratory costs, follow-up costs, adverse event (AE) costs, and costs of end-of-life care. The costs related to healthcare services in the Unites States were inflated to 2018 values based on the Unites States consumer price index (US Department of Labor, 2019). In China, the costs were translated into 2018 Unites States dollars (annual average rate: $1 = CNY 6.8) (National Bureau of Statistics of China, 2019), which were not inflated because the Chinese cost of health remained stable. The Unites States and Chinese cost estimates are shown in Table 1 and Supplementary Appendix Table S1, respectively.

According to the CheckMate-227 trial (Hellmann et al., 2019), nivolumab is given at a dose of 3 mg/kg of body weight every two weeks plus ipilimumab at a dose of 1 mg/kg every six weeks. The assumed mean body weights in the Unites States and China were 70 and 65 kg, respectively (Wu et al., 2017; Wu and Shi, 2020). Treatment continued until disease progression or unacceptable toxicity or, for the immunotherapy regimens, until two years of follow-up (Hellmann et al., 2019). The prices of ipilimumab and nivolumab in the Unites States (average wholesale price) were derived from CMS (CMS, 2019). Because the wholesale price was lower than the retail price (Curtiss et al., 2010), the vial price of nivolumab and ipilimumab was decreased by 17% to account for Unites States contract pricing, as reported in a previous study by Hornberger and others (Hornberger et al., 2015). We also checked its impact in the sensitivity analysis. The cost related to cytotoxic chemotherapy for untreated metastatic NSCLC was $24,437 per patient regardless of histology (Yu et al., 2018. For nonsquamous NSCLC, the cost related to maintenance chemotherapy was $5,887 per three-week chemotherapy cycle (Yu et al., 2018). The costs of chemotherapy infusion in the first hour and additional hour were $148 and $33, and the subsequent infusion per hour was $70 (Insinga et al., 2018; Insinga et al., 2019). The average one-week costs of disease management (excluding drug, drug administration, and AE-related costs) in the PFD and PD states were stratified by survival years following the initiation of first-line treatment (Insinga et al., 2018; Insinga et al., 2019). The average weekly costs of disease management (excluding drug, drug administration, and AE related costs) in the PFD and PD states were estimated from an analysis of 2013 SEER Medicare data for Stage 4 non-squamous NSCLC patients. The costs related to subsequent therapies applied following the discontinuation of initial trial treatments, managing grade ≥3 AEs, disease management, and terminal care during the last 30 days of life were extracted from the literature (Insinga et al., 2018; Insinga et al., 2019). The Chinese cost data were collected from our previous reports (Chai et al., 2020; Li et al., 2020). We assumed that vial wastage is not permitted. This assumption was examined in the sensitivity analysis.

As previous studies have done (Insinga et al., 2018; Insinga et al., 2019), a time-to-death approach, reflecting the decline in cancer patients’ quality of life, was used for modeling utilities. The utility scores for the ≥360, 180 to <360, 30 to <180, and <30 days time-to-death categories were estimated on the basis of EuroQOL-5D (EQ-5D) 3-level utility data (Table 1).

### Analysis

The main endpoint in the base-case analysis was the incremental cost-effectiveness ratio (ICER), which was estimated as the incremental cost per additional quality-adjusted life-year (QALY) gained between the two alternatives. Cost and QALYs were discounted at an annual rate of 3% in the United States and 5% in China (Sanders et al., 2016; 2011), respectively. We also estimated the incremental net-health benefit (INHB) based on the following formula: INHB(λ) = (μ_E1_ - μ_E0_) - (μ_C1_- μ_C0_)/λ = ΔE - ΔC/λ, where μ_Ci_ and μ_Ei_ are the cost and effectiveness of the new option (i = 1) or old option (i = 0), respectively, and λ is the willingness-to-pay threshold in the United States ($100,000/QALY) and China ($27,351/QALY) (Craig and Black, 2001; Stinnett and Mullahy, 1998; 2011), respectively. Subgroup analyses were conducted in the subgroups as implied in the CheckMate-227 trial, where the INHBs of nivolumab plus ipilimumab vs. chemotherapy were calculated at the lower, mean and upper estimates of the HRs of OS. In the subgroup analysis, we assumed that only the HRs of OS changed.

One-way sensitivity analyses were performed where varied values of each parameter within its range at a specific time were used to examine the effect of these parameters on the ICER. The ranges were derived from the reported or estimated 95% confidence intervals; when reported data were not available, a range of ±25% of the base-case value was used (Table 1). In the one-way sensitivity analysis, we fixed the survival distributions of chemotherapy and adjusted the PFS and OS of the intervention arm by adopting the hazard ratios between nivolumab plus ipilimumab and chemotherapy. Probabilistic sensitivity analyses were carried out where 1,000 Monte Carlo repetitions were generated by sampling all parameters simultaneously during each repetition from the following distributions: gamma distribution for the cost parameters, log-normal distribution for hazard ratios, and beta distribution for the probability, proportion, and preference value parameters. In the probabilistic sensitivity analysis, each parameter in the survival distribution was first sampled based on their expected values, and a variance-covariance matrix was used to calculate the survival probabilities. A cost-effectiveness acceptability curve (CEAC) was constructed, which represents the probability that a strategy is cost-effective compared to the alternative at a range of willingness-to-pay thresholds.

## Results

### Base-Case and Subgroup Analyses

In comparison with chemotherapy, nivolumab plus ipilimumab produced an incremental 1.260 QALYs and 1.787 expected overall life years with an incremental cost of $95,617, which led to an ICER of $75,871/QALY and INHB of 0.304 QALY at the threshold of $100,000/QALY in the United States setting (Table 2). In China, the ICER of nivolumab plus ipilimumab vs. chemotherapy was $59,773/QALY, and the INHB was -1.312 QALY at the threshold of $27,351/QALY.

**TABLE 2:** Summary of cost ($) and outcome results in base-case analysis.

Strategy	Cost	Progression-free LYs	Overall LYs	QALYs	Incremental cost per QALY^a^	INHB^a^
In the context of United States						
Standard chemotherapy (reference strategy)	223,007	0.618	1.971	1.572	NA	NA
Nivolumab plus ipilimumab strategy	318,624	1.269	3.758	2.832	75,871	0.304
In the context of China						
Standard chemotherapy (reference strategy)	36,593	0.617	1.960	1.517	NA	
Nivolumab plus ipilimumab strategy	102,771	1.260	3.695	2.624	59,773	−1.312

a Comparing with reference strategy.

**FIGURE 2:**
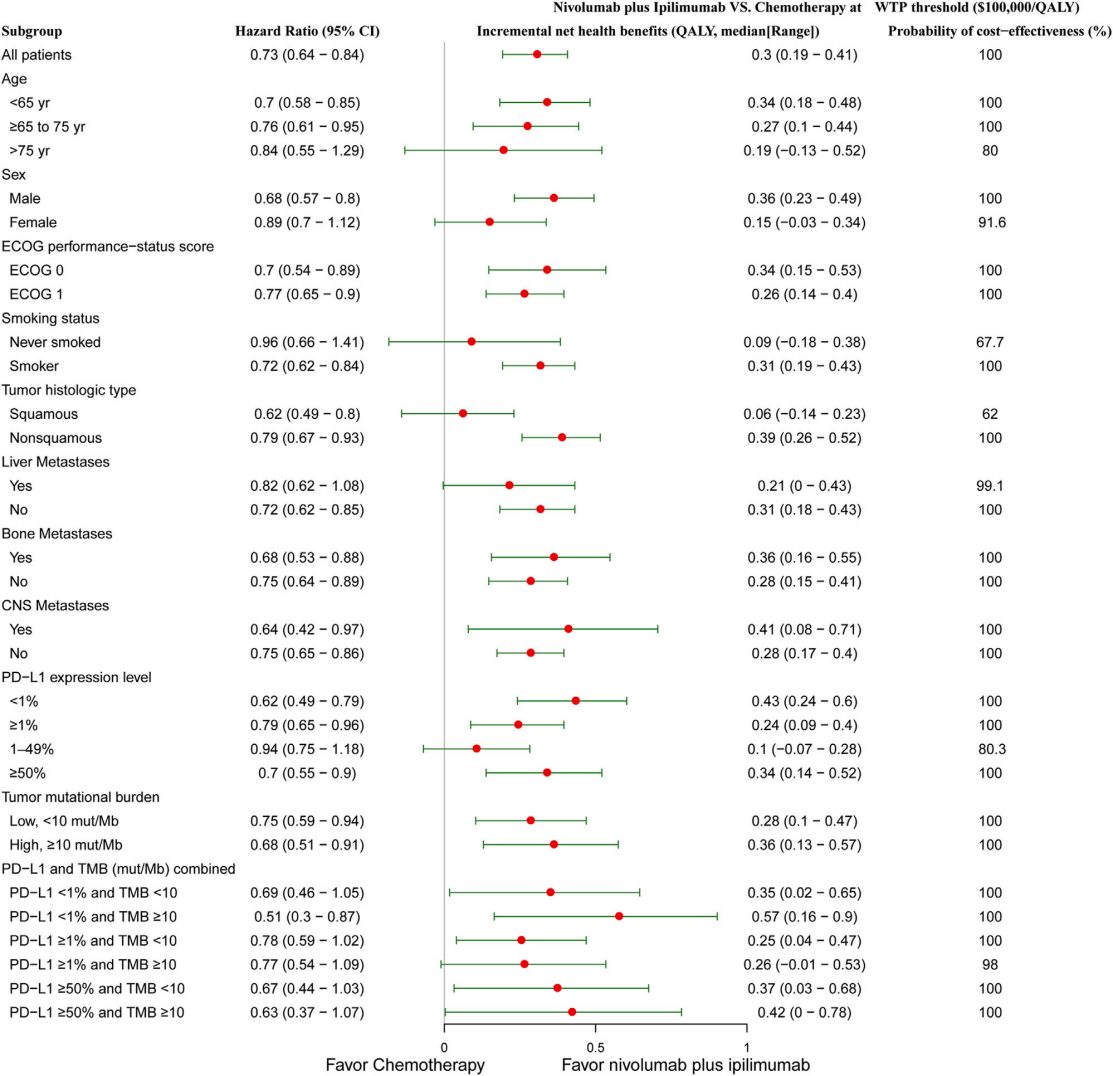
Subgroup analysis of incremental net health benefits (INHB) and probabilities of cost-effectiveness by varying the hazard ratios (HRs) of OS in the context of the United States. The vertical line indicates the point of no effect (INHB = 0), the red circle indicates the median INHB, and the green bar indicates the ranges of INHB adjusted by the HRs.

Subgroup analysis by varying the HRs of OS in the United States found that nivolumab plus ipilimumab presented positive INHBs in all subgroups (Figure 2). The INHBs in the subgroups for the health benefit varied from 0.06 (range: 0.14 to 0.23, probabilities of cost-effectiveness: 62%) in patients with squamous tumors to 0.57 (range: 0.16 to 0.9, probabilities of cost-effectiveness: 100%) in cancer with PD-L1 <1% and tumor mutational burden ≥10 mut/Mb. In the Chinese context, all subgroups resulted in negative INHBs (Supplementary Appendix Figure S3).

### Sensitivity Analyses

**FIGURE 3:**
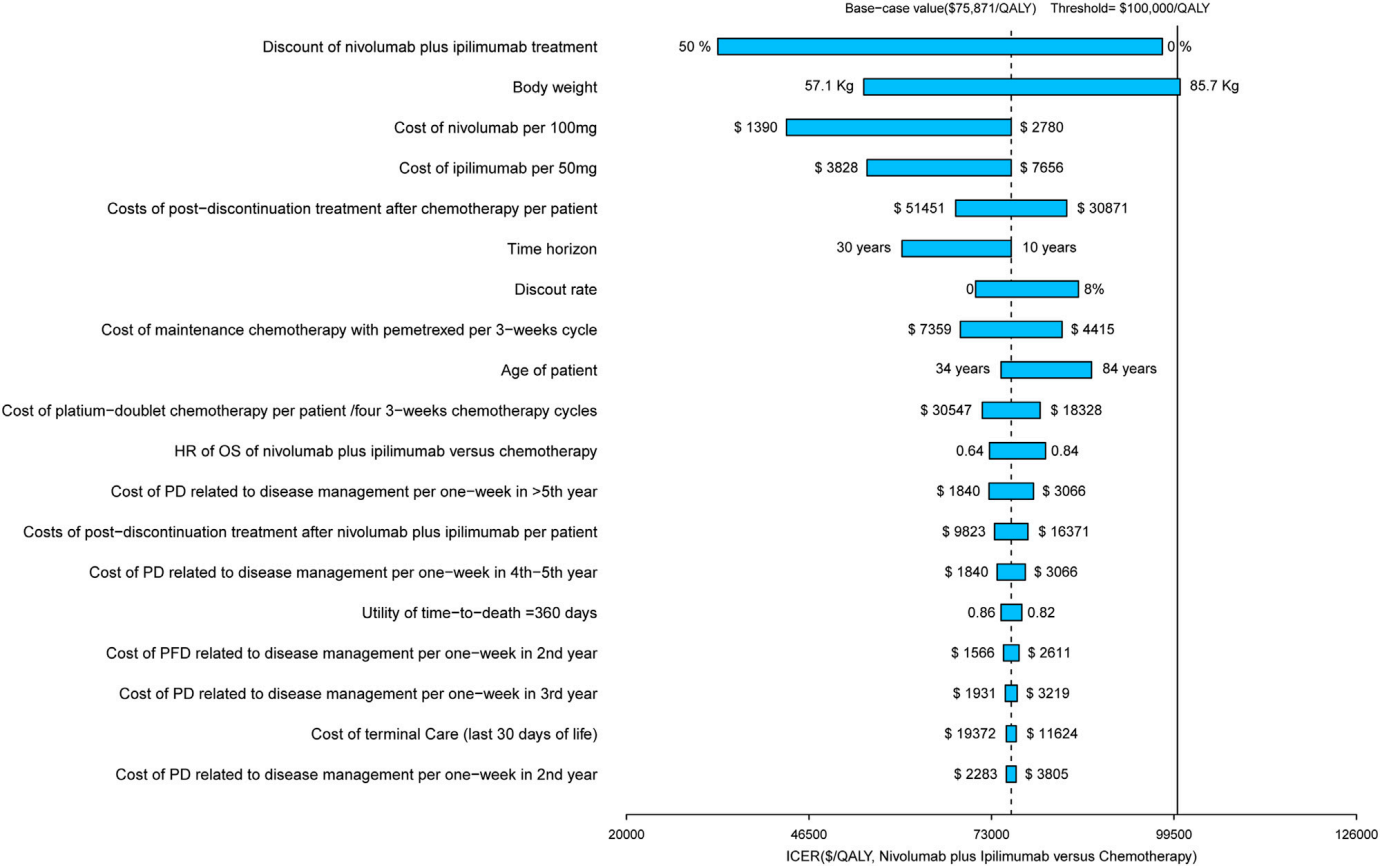
Tornado diagram of one-way sensitivity analyses of nivolumab plus ipilimumab vs. chemotherapy in the context of United States.

The results of the one-way sensitivity analysis are presented in the Tornado diagram (Figure 3), which indicated that the discount of the prices of nivolumab plus ipilimumab treatment played a vital role in model outcomes in the United States. When its values used the lower and upper boundaries, the ICERs of nivolumab plus ipilimumab adjusted from reflected $33,257/QALY to $97,823/QALY respectively. Other considerable parameters that the model was sensitive to included the body weight and the cost of nivolumab and ipilimumab. The rest of the parameters, such as the cost and disutilities associated with managing ADRs, had a medium and small impact on the outcome. In general, the model outcomes were robust to the adjustment of parameters. With the long time horizon, the nivolumab plus ipilimumab therapy would become more cost-effective.

**FIGURE 4:**
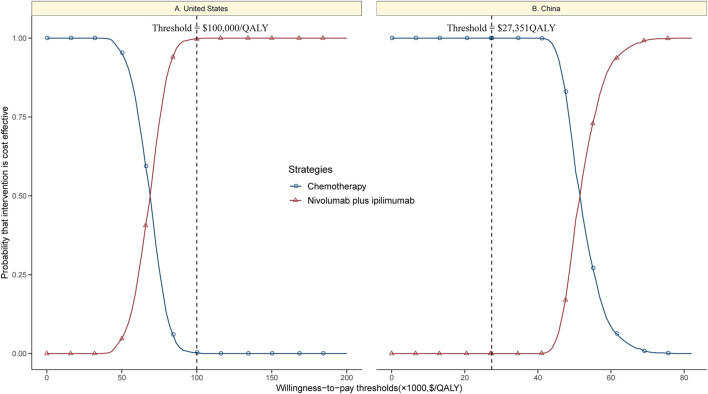
Cost-effectiveness acceptability curves of nivolumab plus ipilimumab vs. chemotherapy in the United States **(A)** and China **(B)**.

The CEAC showed a nearly 99% probability of nivolumab plus ipilimumab and a 1% probability of chemotherapy being a cost-effective strategy at the threshold of $100,000/QALY in the United States setting (Figure 4A). However, nivolumab plus ipilimumab achieved only a nearly 1% probability of cost-effectiveness in the Chinese context (Figure 4B).

In the scenario in which vial wastage was not permitted, the incremental cost of nivolumab plus ipilimumab vs. chemotherapy was $187,137, which led to an ICER of $148,491/QALY.

## Discussion

While oncologists and patients are interested in the clinical benefit of nivolumab plus ipilimumab in the CheckMate-227 trial due to the increasing incidence of lung cancer, the high cost of an anticancer drug can limit its widespread use. Health policymakers and payers assess the economic outcomes of expensive drugs to ensure the ability of patients to access the drug and its sustainability for both national healthcare and reimbursement systems and pharmaceutical companies (Uyl-De and Lowenberg, 2018). Our study addresses this emergent need for the economic assessment of nivolumab plus ipilimumab. Based on the results of the CheckMate-227 trial, our analysis demonstrated nivolumab plus ipilimumab for advanced NSCLC to be preferred for WTP thresholds higher than $75,871 per QALY. This result is generally robust, as shown by the results of both the one-way and probabilistic sensitivity analyses. At a threshold of $100,000/QALY, all subgroups were favored for nivolumab plus ipilimumab because of its positive trend of gaining incremental net health benefits compared to chemotherapy. It should be noted that nivolumab plus ipilimumab showed more favorable economic outcomes in non-squamous tumors than in squamous tumors, although immunotherapy had a better trend of prognosis. The potential reason is that maintenance chemotherapy is considered in nonsquamous tumors, which substantially augments the cost of chemotherapy in nonsquamous tumors compared with squamous tumors. The recent two economic analyses showed the opposite results (Hu et al., 2020; Courtney et al., 2021), which might be led by the different gained health outcomes. However, nivolumab plus ipilimumab is not a cost-effective option in the Chinese context because its ICER exceeded the local threshold of $27,351/QALY. A potential reason for this could be the relatively lower costs related to chemotherapy and the higher costs related to nivolumab plus ipilimumab treatment. Based on our estimation for achieving the Chinese cost-effectiveness threshold, a 64% discount of the cost of nivolumab plus ipilimumab in the deterministic sensitivity analysis could push this regimen to be cost-effective in the Chinese context.

The findings of the one-way sensitivity analysis indicated that body weight is a substantial model input because nivolumab and ipilimumab are administered based on body weight. This result suggested that nivolumab plus ipilimumab would become unfavorable in overweight patients because more nivolumab and ipilimumab would be needed. Because significant wastage has been associated with body size dosing of monoclonal antibodies, dosing strategies without compromising exposure and efficacy should be adopted to reduce the wastage (Ogungbenro et al., 2018). This finding could also be supported by the costs of nivolumab and ipilimumab, which were also found to be two important influential factors. When the unit costs of nivolumab and ipilimumab are discounted by 50%, the ICER for nivolumab plus ipilimumab would be lower than $50,000/QALY in the United States. To help bring down their relatively high prices, the United States and Chinese governments have considered referencing the prices in other countries (Dyer, 2018). Once it is enacted or implemented, this initiative might lead to a reduction in the prices of nivolumab and ipilimumab and achieve more favorable economic outcomes.

This study has several strengths. First, to the best of our knowledge, this is the first study to assess the cost-effectiveness of nivolumab plus ipilimumab therapy in advanced NSCLC by incorporating the latest clinical data through a modeling technique. Monotherapy blockade of PD-1 or such treatment in combination with chemotherapy is becoming popular in advanced NSCLC. However, the economic outcome of the ICI combination of an anti-PD-1 antibody and a CTLA-4 antibody for advanced NSCLC is death. Second, the current analysis examined the economic outcomes of 29 subgroups prespecified by the CheckMate-227 trial. Economic information on each of the subgroups would be helpful for physicians and patients when they have to make a treatment decision the patient will be covering out of pocket. Third, the current analysis examined the economic outcomes in both the United States and China, which are representative of high-income and middle-income countries, respectively. Our findings could be transferred to other high-income and middle-income regions.

There are several limitations in this analysis. First, due to the absence of a head-to-head study, we did not include other ICIs as first-line treatments, such as pembrolizumab and atezolizumab, which have shown favorable health benefits as first-line treatments in combination with chemotherapy or monotherapy (Reck et al., 2016; Mok et al., 2019; Reck et al., 2019; West et al., 2019). The present study needs to be revised when direct comparison data becomes available. Second, health outcomes beyond the observation time of the CheckMate-227 study were assumed through the fitting of parametric survival functions to the PFS and OS data of the trial, which could introduce uncertainty into the results, although we validated the predicted and observed survival data. Third, this analysis did not consider the budget impact of using nivolumab and ipilimumab on society. Because nearly 64,901 new NSCLC patients annually would be eligible for 17.9 first-line treatment cycles of ICI treatment (Goldstein et al., 2017), the first-line prescription of nivolumab plus ipilimumab might substantially increase the financial burden on society. Finally, the costs of managing grade 1/2 AEs were not included in this study, which might overestimate the economic results of nivolumab plus ipilimumab. This weakness may not be a major one, as implied by the findings in the one-way sensitivity analysis, which indicated that the costs related to AEs only have a tiny impact.

In summary, this evaluation demonstrated that nivolumab plus ipilimumab was a cost-effective option for patients with advanced NSCLC harboring no EGFR or ALK mutations from a United States payer perspective. However, in a middle-income country, such as China, nivolumab plus ipilimumab should be considered only when its costs can be substantially reduced. These findings might be helpful for physicians and decision-makers.

## Data Availability

The original contributions presented in the study are included in the article/Supplementary Material, further inquiries can be directed to the corresponding author.
