# Supplementary material for: Cost-Effectiveness of Nivolumab Plus Ipilimumab as First-Line Therapy in Advanced Non–small-cell Lung Cancer
Source: Front Pharmacol. 2021 Jul 5;12:573852. doi: 10.3389/fphar.2021.573852 (PMC8287729; doi:10.3389/fphar.2021.573852)
Supplement: Supplementary file 1 [file DataSheet1.PDF]

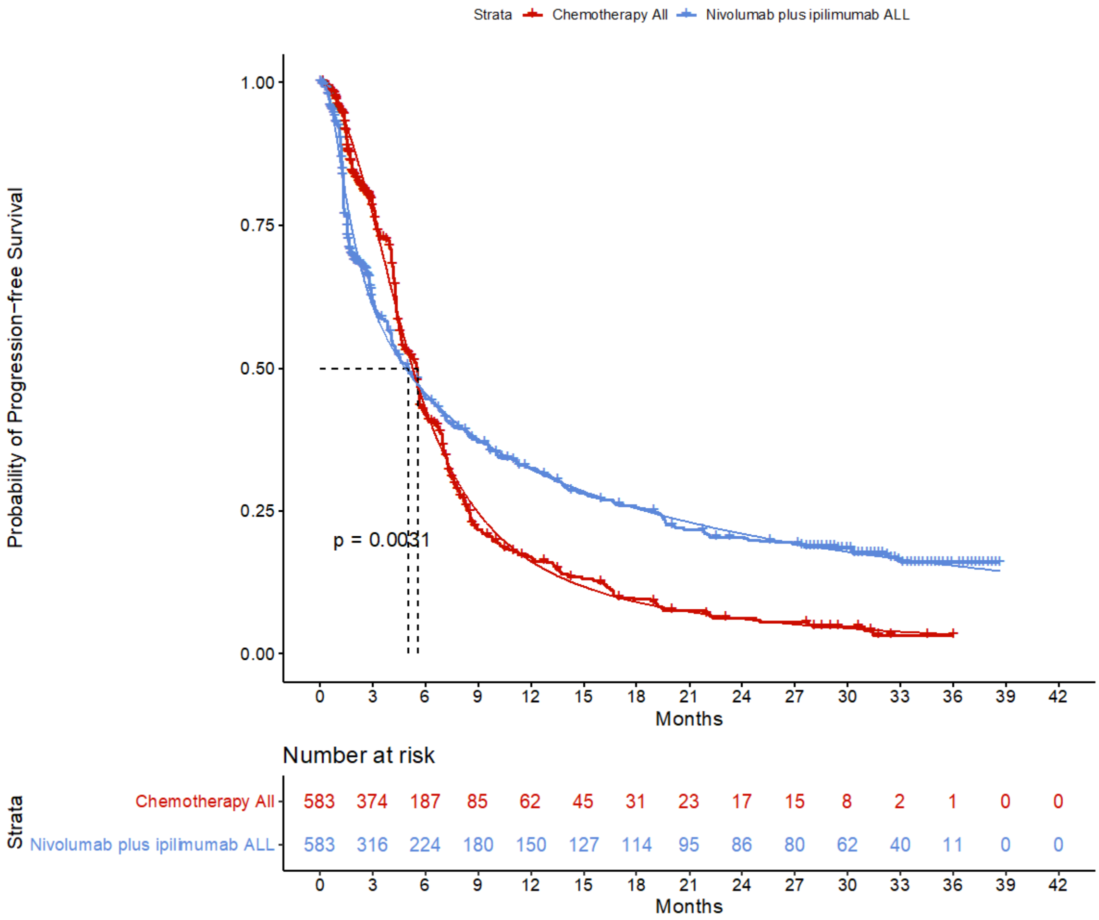

Appendix Figure 1: The replicated Kaplan–Meier PFS curves of nivolumab plus ipilimumab and chemotherapy in the CheckMate-227 trial. The smooth lines indicated the survival curves predicting their corresponding best survival distributions.

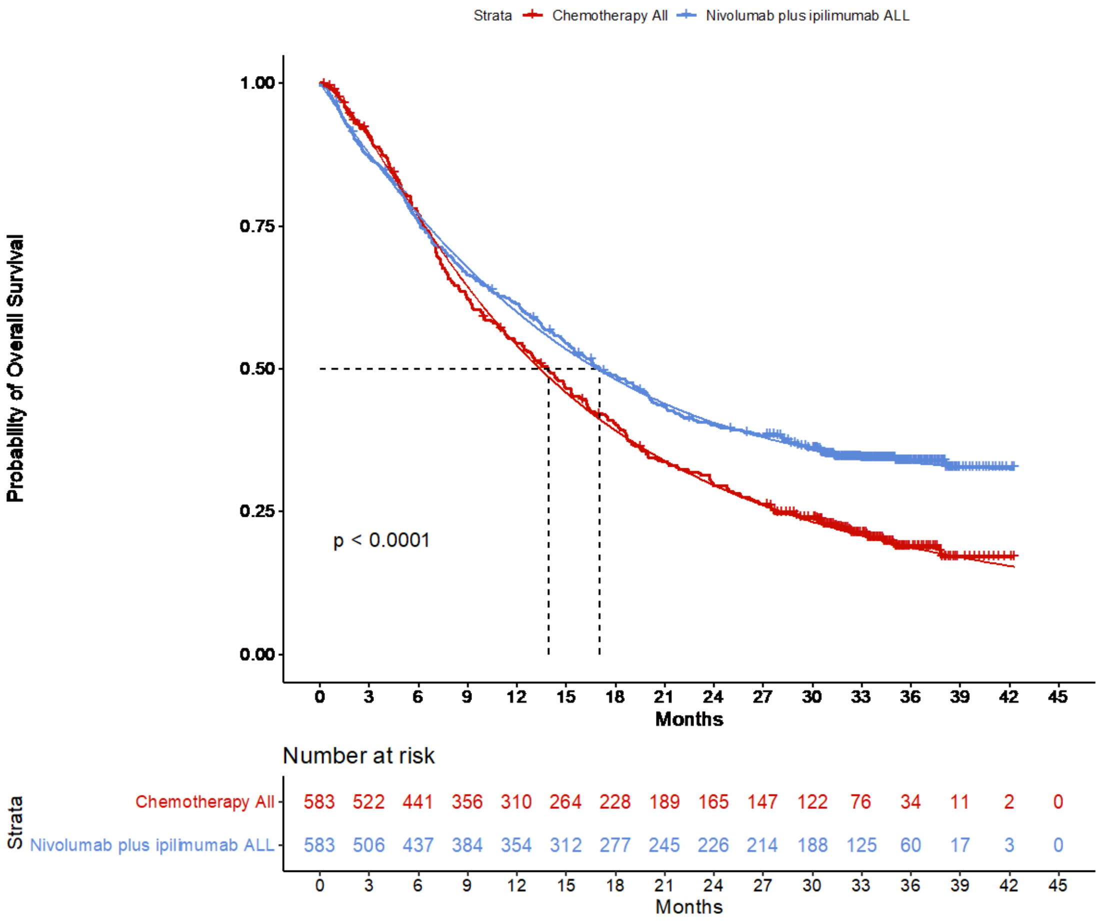

Appendix Figure 2: The replicated Kaplan–Meier OS curves of nivolumab plus ipilimumab and chemotherapy in the CheckMate-227 trial. The smooth lines indicated the survival curves predicting their corresponding best survival distributions.

Nivolumab plus Ipilimumab VS. Chemotherapy at WTP threshold ($100,000/QALY)

Subgroup	Hazard Ratio (95% CI)	Incremental net health benefits (QALY, median[Range])	Probability of cost−effectiveness
All patients	0.73 (0.64 − 0.84)	−1.97 (−2.07 − −1.88)	0
Age			
<65 yr	0.7 (0.58 − 0.85)	−1.94 (−2.08 − −1.81)	0
=65 to 75 yr	0.76 (0.61 − 0.95)	−2 (−2.17 − −1.85)	0
>75 yr	0.84 (0.55 − 1.29)	−2.07 (−2.41 − −1.78)	0
Sex			
Male	0.68 (0.57 − 0.8)	−1.92 (−2.04 − −1.8)	0
Female	0.89 (0.7 − 1.12)	−2.12 (−2.29 − −1.94)	0
ECOG performance−status score			
ECOG 0	0.7 (0.54 − 0.89)	−1.94 (−2.12 − −1.77)	0
ECOG 1	0.77 (0.65 − 0.9)	−2.01 (−2.12 − −1.89)	0
Smoking status			
Never smoked	0.96 (0.66 − 1.41)	−2.17 (−2.49 − −1.9)	0
Smoker	0.72 (0.62 − 0.84)	−1.96 (−2.07 − −1.86)	0
Tumor histologic type			
Squamous	0.62 (0.49 − 0.8)	−1.86 (−2.04 − −1.71)	0
Nonsquamous	0.79 (0.67 − 0.93)	−2.03 (−2.15 − −1.91)	0
Liver Metastases			
Yes	0.82 (0.62 − 1.08)	−2.06 (−2.26 − −1.86)	0
No	0.72 (0.62 − 0.85)	−1.96 (−2.08 − −1.86)	0
Bone Metastases			
Yes	0.68 (0.53 − 0.88)	−1.92 (−2.11 − −1.76)	0
No	0.75 (0.64 − 0.89)	−1.99 (−2.12 − −1.88)	0
CNS Metastases			
Yes	0.64 (0.42 − 0.97)	−1.88 (−2.18 − −1.62)	0
No	0.75 (0.65 − 0.86)	−1.99 (−2.09 − −1.89)	0
PD−L1 expression level			
<1%	0.62 (0.49 − 0.79)	−1.86 (−2.03 − −1.71)	0
=1%	0.79 (0.65 − 0.96)	−2.03 (−2.17 − −1.89)	0
1–49%	0.94 (0.75 − 1.18)	−2.16 (−2.33 − −1.99)	0
=50%	0.7 (0.55 − 0.9)	−1.94 (−2.12 − −1.78)	0
Tumor mutational burden			
Low, <10 mut/Mb	0.75 (0.59 − 0.94)	−1.99 (−2.16 − −1.83)	0
High, =10 mut/Mb	0.68 (0.51 − 0.91)	−1.92 (−2.13 − −1.73)	0
PD−L1 and TMB (mut/Mb) combined			
PD−L1 <1% and TMB <10	0.69 (0.46 − 1.05)	−1.93 (−2.24 − −1.67)	0
PD−L1 <1% and TMB =10	0.51 (0.3 − 0.87)	−1.73 (−2.1 − −1.46)	0
PD−L1 =1% and TMB <10	0.78 (0.59 − 1.02)	−2.02 (−2.22 − −1.83)	0
PD−L1 =1% and TMB =10	0.77 (0.54 − 1.09)	−2.01 (−2.27 − −1.77)	0
PD−L1 =50% and TMB <10	0.67 (0.44 − 1.03)	−1.91 (−2.23 − −1.65)	0
PD−L1 =50% and TMB =10	0.63 (0.37 − 1.07)	−1.87 (−2.26 − −1.56)	0

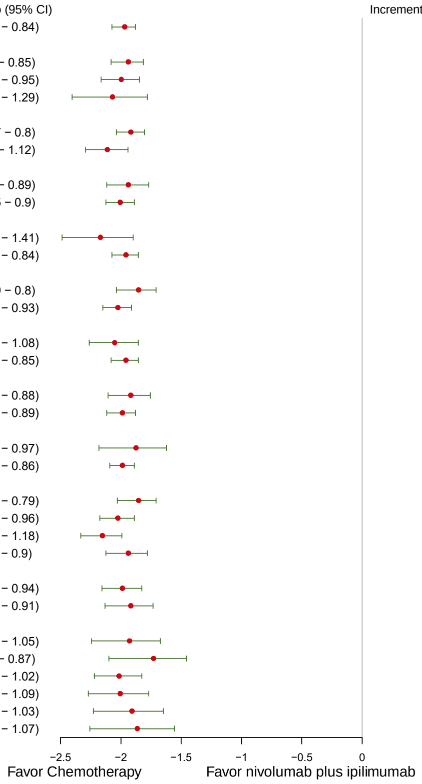

Appendix Figure 3: Subgroup analysis of incremental net health benefits (INHB) and probabilities of cost-effectiveness by varying the hazard ratios (HRs) of OS in the Chinese context. The vertical line indicates the point of no effect (INHB = 0), the red circle indicates the median INHB, and the green bar indicates the ranges of INHB adjusted by the HRs.

Appendix Table1: Cost ($) estimates in the Chinese context*.

Paramters	Expected value	Range	Distribution
Ipilimumab per 50mg	4,655	2,328 - 7,324	Fixed
Nivolumab per 100mg	1,362	681 - 1,362	Fixed
Platium-doublet chemotherapy per patient /four 3-weeks chemotherapy cycles	2,747	1,348 - 5,355	Gamma: α= 62891, λ= 0.297
Maintenance chemotherapy with pemetrexed per 3-weeks cycle	1,753	1,001 - 3,962	Gamma: α= 27663, λ= 0.431
Post-discontinuation treatment in nivolumab plus ipilimumab treatment	3,900	2,227 - 8,815	Gamma: α= 61539, λ= 0.431
Post-discontinuation treatment in standard chemotherapy treatment	12,258	6,998 - 27,703	Gamma: α= 193404, λ= 0.431
Disease management in PFD state per one-week in 1st year	1,173	669 - 2,650	Gamma: α= 18503, λ= 0.431
Disease management in PFD state per one-week in 2nd year	1,173	669 - 2,650	Gamma: α= 18503, λ= 0.431
Disease management in PFD state per one-week in 3rd year	622	355 - 1,406	Gamma: α= 9813, λ= 0.431
Disease management in PFD state per one-week in 4th to 5th year	275	157 - 620	Gamma: α= 4331, λ= 0.431
Disease management in PFD state per one-week in after 5 years	228	130 - 515	Gamma: α= 3597, λ= 0.431
Disease management in PD state per one-week in 1st year	100	57 - 227	Gamma: α= 1583, λ= 0.431
Disease management in PD state per one-week in 2nd year	1,294	739 - 2,925	Gamma: α= 20417, λ= 0.431
Disease management in PD state per one-week in 3rd year	906	517 - 2,049	Gamma: α= 14302, λ= 0.431
Disease management in PD state per one-week in 4th to 5th year	767	438 - 1,733	Gamma: α= 12101, λ= 0.431
Disease management in PD state per one-week in after 5 years	730	417 - 1,651	Gamma: α= 11525, λ= 0.431
Managing AE (grade ≥3) per patient related to ICI treatment	446	255 - 1,009	Gamma: α= 7043, λ= 0.431
Managing AE (grade ≥3) per patient related to chemotherapy	375	214 - 847	Gamma: α= 5916, λ= 0.431
Terminal Care (last 30 days of life)	4,616	2,635 - 10,431	Gamma: α= 72821, λ= 0.431

Abbreviations: AE, adverse event; PFS, progression-free survival;PFD, progression-free disease; PD, progressed disease; OS, overall survival.

* Chinese costs were translated into US dollars (US $ 1 = CNY ￥6.8).
